# Characterization of Ubiquitin-Activating Enzyme Uba1 in the Nucleus by Its Mammalian Temperature-Sensitive Mutant

**DOI:** 10.1371/journal.pone.0096666

**Published:** 2014-05-07

**Authors:** Kimihiko Sugaya, Yoshie Ishihara, Sonoe Inoue, Hideo Tsuji

**Affiliations:** 1 Research Center for Radiation Protection, National Institute of Radiological Sciences, Chiba, Japan; 2 Fukushima Project Headquarters, National Institute of Radiological Sciences, Chiba, Japan; University of Minnesota, United States of America

## Abstract

Temperature-sensitive (ts) CHO-K1 mutant tsTM3 exhibits chromosomal instability and cell-cycle arrest in the S to G_2_ phases with decreased DNA synthesis at the nonpermissive temperature, 39°C. Previously, complementation tests with other mutants showed that tsTM3 harbors a genetic defect in the ubiquitin-activating enzyme Uba1. Sequence comparison of the *Uba1* gene between wild-type and mutant cells in this study revealed that the mutant phenotype is caused by a G-to-A transition that yields a Met-to-Ile substitution at position 256 in hamster Uba1. The ts defects in tsTM3 were complemented by expression of the wild-type Uba1 tagged with green fluorescent protein. Expression of the Uba1 primarily in the nucleus appeared to rescue tsTM3 cells. Incubation at 39°C resulted in a decrease of nuclear Uba1 in tsTM3 cells, suggesting that loss of Uba1 in the nucleus may lead to the ts defects. Analyses with the fluorescent ubiquitination-based cell cycle indicator revealed that loss of function of Uba1 leads to failure of the ubiquitin system in the nucleus. Incubation at 39°C caused an increase in endogenous geminin in tsTM3 cells. A ts mutation of *Uba1* found in tsTM3 cells appears to be a novel mutation reflecting the important roles of Uba1 in nucleus.

## Introduction

The ubiquitination process requires the coordinated action of three enzymes: ubiquitin (Ub) activating enzyme (E1), Ub conjugating enzyme (E2) and Ub ligase (E3) [1]. E1 catalyzes the initial step in the Ub conjugation pathway. Ub is activated during this reaction and serves as a substrate for the subsequent enzymes in the conjugation cascade. We now know that ubiquitination participates not only in the proteolytic function but also in many non-proteolytic reactions with crucial roles in cell metabolisms [2]. For example, fluorescence ubiquitination-based cell cycle indicator (Fucci) enabled us to examine cell division within living cells by the Ub-proteasome system [3]. In mammalian cells, there are dozens of E2s and several hundred E3s, and both define families of proteins displaying substrate specificity. However, there are only two E1 enzymes for the entire array of downstream reactions in mammals, Uba1 and Uba6 [4]. *Uba1* encodes canonical E1. Previously, introduction and expression of epitope-tagged Uba1 cDNA constructs revealed that nuclear and cytoplasmic isoforms of Uba1 translate from first and second ATG (Met at 41) codons: E1a, localized predominantly in the nucleus, and E1b, localized in the cytoplasm, respectively [5]. To avoid confusion in terminology, we respectively refer to these two isoforms as Uba1A, defined here as the predominantly nuclear form of Uba1, and Uba1B, defined here as the cytoplasmic form of Uba1, instead of E1a and E1b. Uba6 is required to activate the E2 Use1 (Uba6-specific E2) both in vitro and in vivo [6] and can also activate another ubiquitin-like modifier, FAT10 [7].

To identify genes responsible for the maintenance of chromosome integrity, Tsuji and colleagues isolated 25 temperature-sensitive (ts) mutants from hamster wild-type CHO-K1 cells [8]. Using two of these mutants, we revealed that ts defects in RNA polymerase II and a protein involved in splicing gave rise both to chromosome instability and to cell cycle arrest [9]–[12]. Another ts CHO-K1 mutant, tsTM3, exhibits chromosomal instability and cell-cycle arrest in the S to G_2_ phases with decreased DNA synthesis at the nonpermissive temperature, 39°C. Complementation tests with other mutants showed that tsTM3 did not complement with the Uba1-defective ts mutant ts85 [13] and DNA replication-defective ts mutant ts131b [14], suggesting that these mutants harbor the same genetic defect [8]. From 1980 to 1990, many ts mutants of Uba1 were isolated from several cell lines: ts85 of FM3A [13], ts20 of CHO [15], ts131b of FM3A [14], ts20 of Balb/c 3T3 [16], tsBN75 of BHK21 [17], tsFS20 of FM3A [18], and tsFT5 of FM3A [19]. This unusually high incidence of Uba1 mutations was discussed in terms of Uba1 as a determinant of heat tolerance of cells and the fact that the Uba1 locus is located on the X chromosome [18]. In regard to the connection between Uba1 and human disease, a recent study identified the association of pathogenic mutations in human ***UBA1*** with an early-onset neurodegenerative disorder involving lower motor neurons [20]. It provided evidence that the rare missense and synonymous mutations detected in exon 15 of *UBA1* are associated with X-linked spinal muscular atrophy.

In the present study, to identify the mechanism underlying the tsTM3 phenotypes, we performed sequence analysis of the *Uba1* gene and investigated the relation between a wild-type isoform of Uba1 tagged with green fluorescent protein (GFP) and its localization. Changes of Uba1 in tsTM3 cells during incubation at 39°C were examined by immunoblotting. To analyze ubiquitination activity in the nucleus, we isolated cells expressing Fucci and investigated changes of Fucci with live-cell imaging and Western blotting. Endogenous proteins related to licensing of DNA replication were also examined by Western blotting and by indirect immunolabeling. Finally, we discuss the role of Uba1 in the nucleus.

## Results

### A ts mutation of *Uba1* found in tsTM3

To identify the causative mutation, we determined the sequences of *Uba1* cDNAs from wild-type (CHO-K1) and mutant (tsTM3) cells and deposited the 3174-nucleotide sequence of *Uba1* from CHO-K1 cells as Accession No. AB661372 in the DDBJ, GenBank, and EMBL databases. Comparisons of the deduced amino acid sequence of hamster Uba1 with those of mouse, rat, and human Uba1 revealed 96.9%, 97.9%, and 96.6% homologies, respectively, suggesting its highly conserved gene structure.

**Figure 1:**
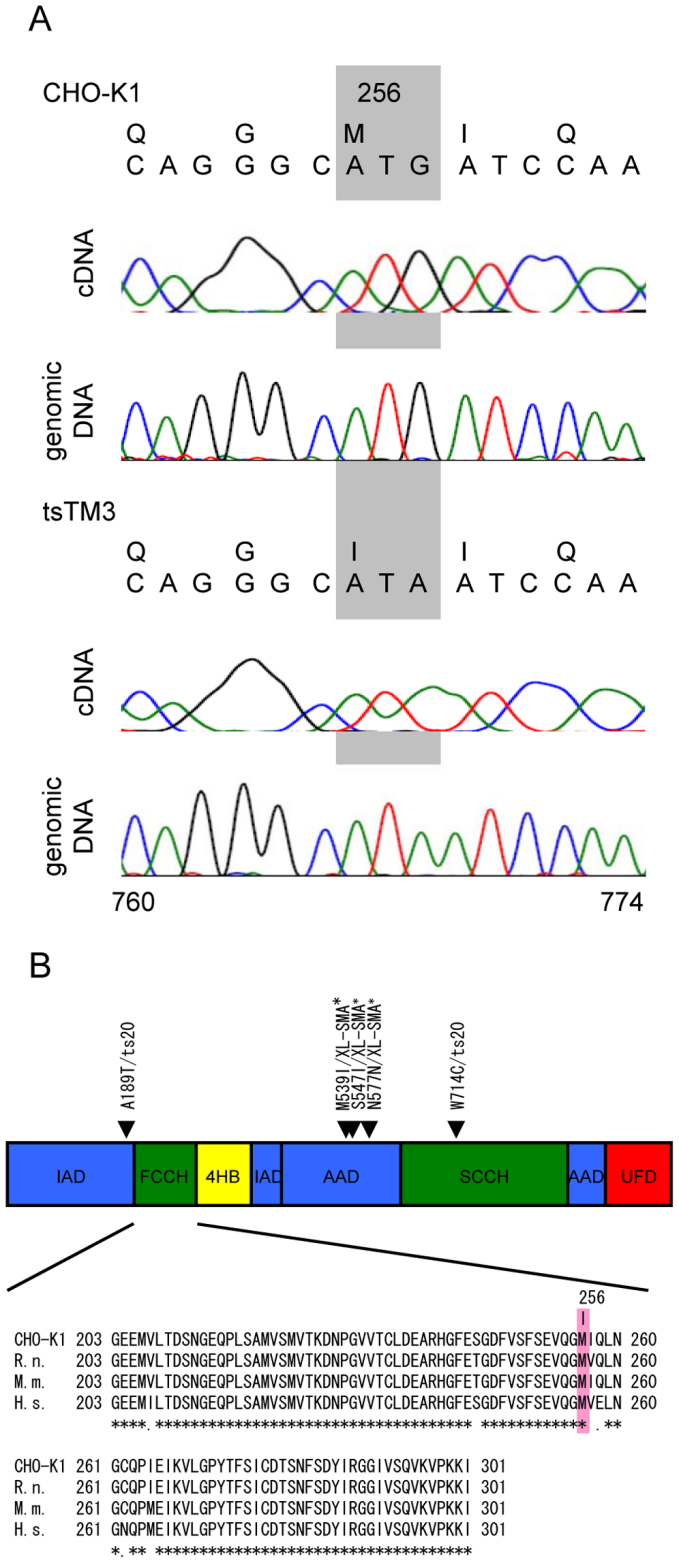
Sequences around the *Uba1* mutation in tsTM3 cells. (A) Sequencing traces obtained with *Uba1* DNA (prepared by reverse transcription of pools of mRNA or from genomic DNA) from wild-type CHO-K1 and mutant tsTM3 cells. The wild type contains a G at nucleotide 768 of *Uba1*, whereas the mutant contains an A. The G-to-A transition at nucleotide 768 converts Met to Ile at amino acid 256. (B) Structure of mammalian Uba1. Colored boxes represent four functional domains, adenylation domains (IAD and AAD), catalytic cysteine half-domains (FCCH and SCCH), four-helix bundle domain (4HB), and C-terminal ubiquitin-fold domain (UFD). Locations of the mutation found in ts mutant ts20 [21] and in X-linked spinal muscular atrophy (XL-SMA) [20] are shown above the diagram. Alignments were made with CLUSTAL W [37] relative to positions 203–301 of hamster Uba1 (Accession No. AB661372) with sequences from *Homo sapiens* (H. s.; NCBI Gene ID: 7317), *Mus musculus* (M. m.; ID: 22201), and *Rattus norvegicus* (R. n.; ID: 314432). Asterisks indicate identical residues.

Sequence comparison between wild-type and mutant revealed a clear difference: a G-to-A transition at nucleotide 768 of *Uba1* in tsTM3 cells (Fig. 1A). This difference was confirmed with polymerase chain reaction (PCR) amplification of the relevant genomic regions in DNAs from wild-type and mutant cells (Fig. 1A). This G-to-A transition results in a Met-to-Ile substitution at position 256 in the protein (Fig. 1A). The substituted methionine is conserved in mammals and *Danio rerio*, *Drosophila melanogaster*, *Anopheles gambiae*, *Schizosaccharomyces pombe*, *Arabidopsis thaliana*, and *Oryza sativa*, suggesting that this is an important residue. Uba1 consists of four functional domains: adenylation domains (IAD and AAD), the first and second catalytic cysteine half-domains (FCCH and SCCH, respectively), the four-helix bundle domain (4HB), and the C-terminal Ub-fold domain (UFD) [21]. The methionine residue mutated in tsTM3 cells was localized in the FCCH domain (Fig. 1B).

### Significant decrease in Uba1 in tsTM3 cells incubated at 39°C

**Figure 2:**
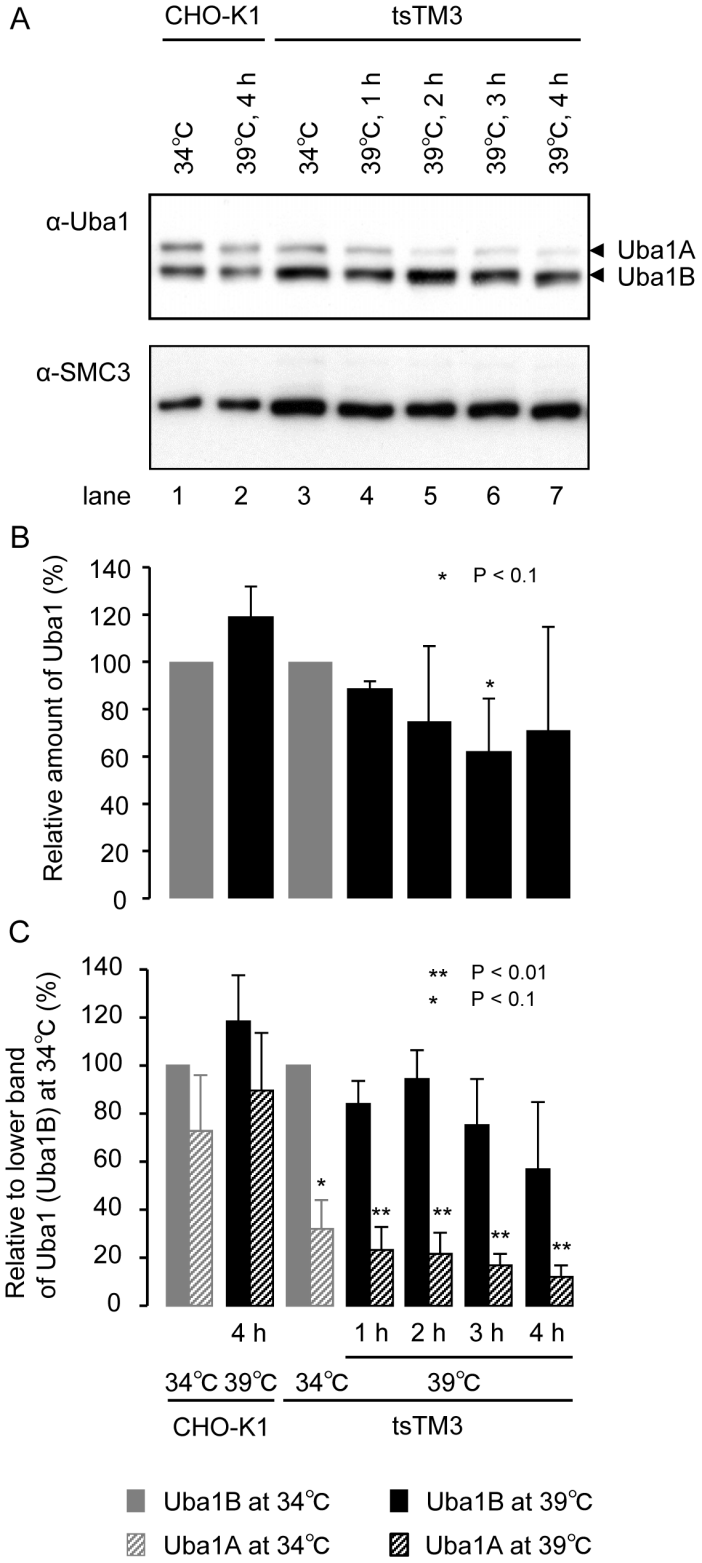
Temperature-dependent reduction in the amount of Uba1 in tsTM3 cells. (A) Western blot analysis of Uba1 in tsTM3 cells. Wild-type CHO-K1 or ts mutant tsTM3 cells were grown at 34°C or incubated at 39°C for the times shown in the figure and lysed. The proteins resolved on acrylamide gels and were detected by immunoblotting with an antibody directed against Uba1 (rabbit polyclonal antibody supplied from Rockland). Uba1 exists as two isoforms: Uba1A (∼117 kDa), localized predominantly in the nucleus and Uba1B (∼110 kDa), localized in the cytoplasm. Similar results were obtained from another antibody (rabbit polyclonal from Calbiochem). Only relevant parts of the blot are shown. SMC3 was included as a loading control. Temperature-dependent reduction in the amount of Uba1 was found in tsTM3 cells. (B, C) Quantitative analyses of the amounts of Uba1 in CHO-K1 and tsTM3 cells. Band intensities, like those shown in panel (A), were measured and expressed relative to that of each at 34°C or relative to the lower band at 34°C with standard deviation of at least three experiments. Bands of Uba1B at 34°C in each cell are selected as a control to compare the amount of Uba1 because it appeared to be permanent. *P* values were calculated by Student *t*-test. Significant differences from values at 34°C (B, gray bars; C, solid gray bars) are shown by asterisks. Incubation at 39°C resulted in a significant decrease in Uba1A in tsTM3 cells, although the proportion of Uba1A to Uba1B in mutant cells at 34°C was smaller than that of wild-type CHO-K1.

Wild-type CHO-K1 or ts mutant tsTM3 cells were grown at 34°C or incubated at 39°C for several different times, as shown in Figure 2, and lysed. The proteins resolved on acrylamide gels and were detected by immunoblotting with antibodies directed against Uba1 and SMC3 as a loading control. Uba1 exists as two isoforms: Uba1A (−117 kDa) and Uba1B (−110 kDa). Western blot analysis with anti-Uba1 antibody revealed a small quantity of Uba1A in tsTM3 cells at 39°C, whereas little or no change in the amount of SMC3 was found under the same condition (Fig. 2A). Quantitative analysis of Uba1 revealed that total Uba1 had decreased gradually by 60% relative to that at 34°C after 4 hours incubation at 39°C (Fig. 2B). Especially, incubation at 39°C resulted in a significant decrease in Uba1A in tsTM3 cells, although the proportion of Uba1A to Uba1B in mutant cells at 34°C was smaller than that in wild-type CHO-K1 (Fig. 2C). These results suggest that a critical decrease of Uba1 in the nucleus may lead to the ts defects in tsTM3 cells seen at 39°C.

### Complementation by the expression of wild-type Uba1 tagged with GFP

**Figure 3:**
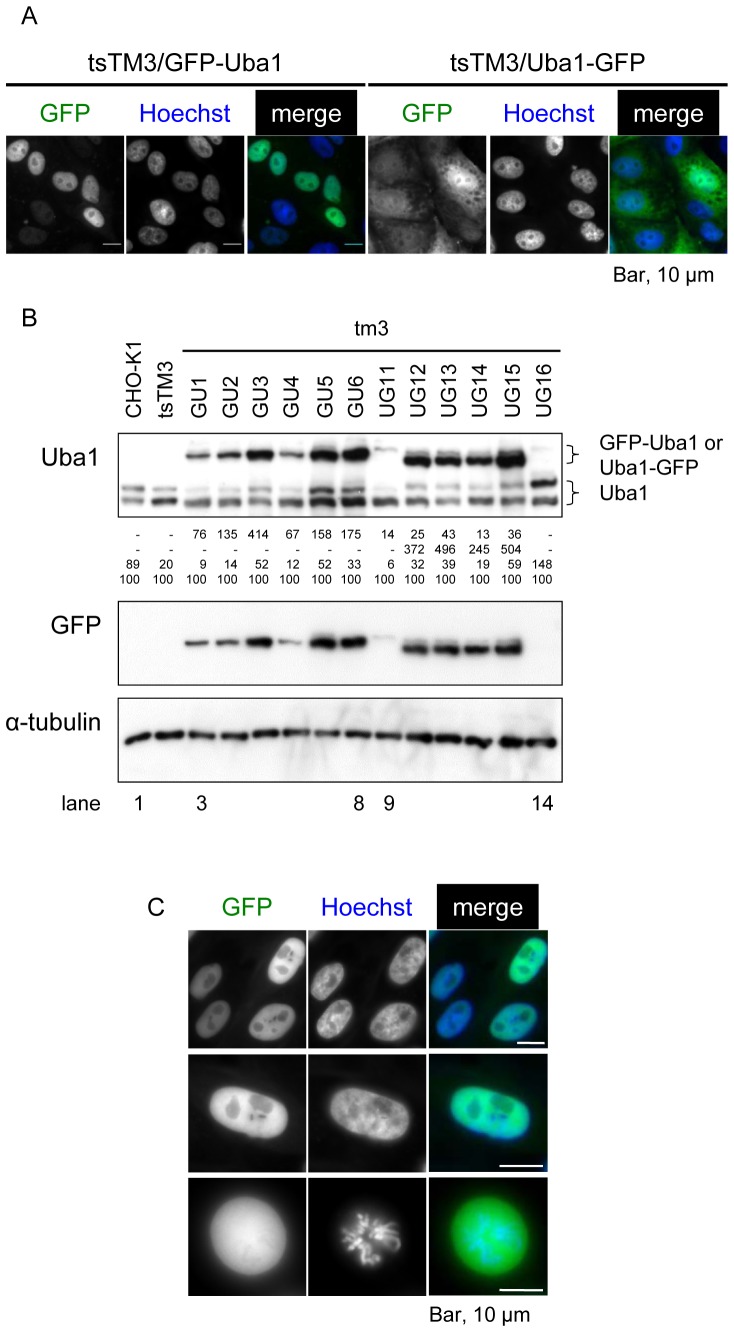
Properties of cell lines expressing Uba1 tagged with green fluorescent protein (GFP). (A) Isoform of Uba1 tagged with GFP and its localization. Cell lines expressing GFP-Uba1 or Uba1-GFP constructs were isolated from the ts mutant cell line, tsTM3. Uba1 tagged with GFP complements deficiencies in tsTM3 cells and allows them to grow normally at 39°C. Cells were counterstained with Hoechst 33342. The fluorescent Uba1 in the GFP-Uba1 derivative is mainly nuclear, whereas the other derivative, Uba1-GFP, contains higher concentrations in both nucleus and cytoplasm. Bar, 10 µm. (B) Western blot analysis of Uba1 tagged with GFP. Cells expressing GFP-Uba1 or Uba1-GFP, as well as parental (tsTM3) and grandparental (CHO-K1) cells were lysed and the proteins resolved on acrylamide gels. Six samples of each distinct GFP clone were analyzed: lanes 3–8, GFP-Uba1; lanes 9–14, Uba1-GFP. Different forms of Uba1 were detected by immunoblotting with antibodies directed against Uba1 and GFP. Only relevant parts of the blot are shown. α-tubulin was included as a loading control. Positions of endogenous and hybrid Uba1 are shown. Band intensities relative to that of the endogenous cytoplasmic form of Uba1 in each of the clones are presented below the blot of Uba1. A derivative of tsTM3 cells, tm3UG16, appears to express very little Uba1-GFP, suggesting the possibility of spontaneous reversion. It is also possible that the rescue depends on a cleaved form of hybrid that resembles the endogenous form but which can no longer be detected via fluorescence. Slightly different migration of bands is observed in tm3UG11. (C) Images of living tsTM3 cells expressing Uba1 tagged with GFP. Cells were counterstained with Hoechst 33342. Upper and middle rows represent living interphase cells expressing the GFP-Uba1 construct. The fluorescent Uba1 in a derivative, tm3GU1, is predominantly in the nucleus. Living mitotic cells (bottom). Bar, 10 µm.

To further verify *Uba1* as the causative gene for tsTM3, we cloned the cDNA of wild-type Uba1 into vectors fused to the amino or the carboxyl terminus with GFP to obtain vectors encoding GFP-Uba1 or Uba1-GFP hybrids. The deficiency of tsTM3 cells was complemented by introducing both hybrid constructs (Fig. 3A). Many clones able to grow at 39°C were obtained, and judging from their growth, observation by microscopy, and levels of expression, we selected six clones of each construct. Different clones expressed different amounts of GFP detectable by fluorescence microscopy; GFP fused to the N-terminus of Uba1 (GFP-Uba1) existed predominantly in the nucleoplasmic region, whereas clones expressing GFP fused to the C-terminus of Uba1 (Uba1-GFP) had brightly fluoresced nuclei and cytoplasm (Fig. 3A). The hybrids complement deficiencies in tsTM3 cells and allow them to grow normally at 39°C, and they enable us to study the dynamics of Uba1 in living cells.

We next determined how much Uba1 tagged with GFP was expressed to rescue tsTM3 cells from incubation at 39°C. Western blot analysis with a rabbit polyclonal antibody raised against Uba1 revealed that the GFP-Uba1 was found as a large form of Uba1 (GFP-Uba1A) that was 0.7∼4.1-fold larger than that of the endogenous cytoplasmic form of Uba1 (Uba1B), and little was found as a small form (Fig. 3B). Uba1-GFP showed both large and small forms, 0.1∼0.4-fold smaller and 2.5∼5.0-fold larger, respectively, than that for the endogenous Uba1B (Fig. 3B). Western blot analysis with a rabbit polyclonal antibody raised against GFP confirmed the single large form of GFP-Uba1 and both the large and small forms of Uba1-GFP (Fig. 3B). To be consistent with the results of fluorescence microscopy, these results suggest that expression of the large form of wild-type Uba1 appeared to rescue tsTM3 cells from incubation at the non-permissive temperature.

Microscopy of living cells revealed that GFP signals in a clone of GFP-Uba1/tsTM3, tm3GU1, were spread in the nucleoplasmic region (Fig. 3C). Surprisingly, the cytoplasmic form of Uba1 may not be required for cell survival. Sparse GFP signals in the cytoplasmic region appear to be consistent with the results obtained by Western blot analysis (Fig. 3B). GFP fusion to the N-terminus of Uba1 may function to block production of the cytoplasmic form. By the middle of mitosis, the condensed chromosomes appeared dark against a bright cytoplasm (Fig. 3C). A clone of GFP-Uba1/tsTM3, tm3GU1, appeared to proceed through mitosis normally at 39°C.

### Complementation by the expression of Uba1 lacking 40 amino acids at the N-terminus

**Figure 4:**
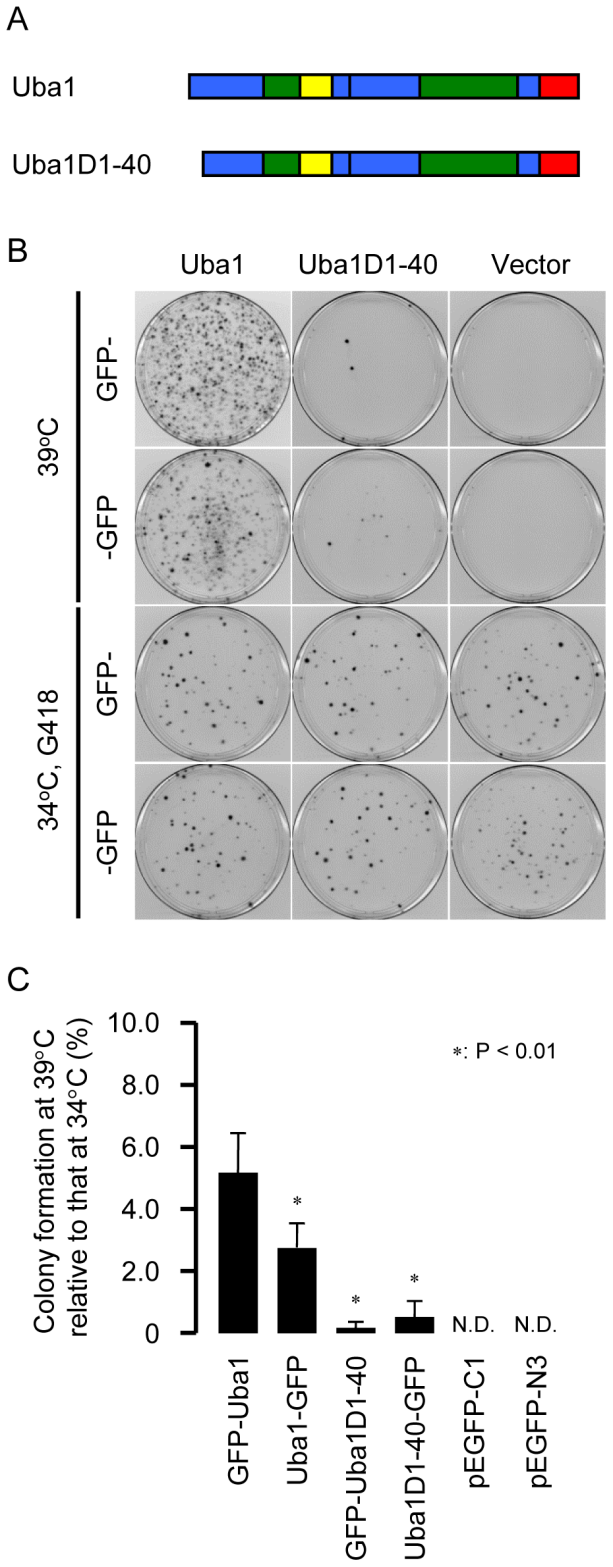
Rescue of tsTM3 cells at 39°C with the expression of full length or truncated forms of Uba1. (A) Diagram of the structures of the full-length and truncated forms of Uba1. Boxes represent the domains of Uba1 in Figure 1B. (B) Photographs of colonies of tsTM3 cells after transfection of 2 µg of plasmid DNAs encoding GFP hybrids of Uba1 or Uba1D1-40 and vectors. After 14 days of incubation at 39°C or 34°C with 400 µg/ml G418, the colonies on the dishes were stained with methylene blue. Cells survived at 39°C after transfection of Uba1 derivatives; however, relatively few cells and no cells grew after transfection of Uba1D1-40 derivatives and vectors, respectively. There was no difference in the colony formation between plasmid DNAs encoding GFP hybrids of Uba1 and Uba1D1-40 after the selection for resistance to G418. (C) Quantitative analysis of colony formation at 39°C. Colonies such as those shown in panel (B) were counted. The colony formation efficiencies of 39°C were normalized to those of 34°C with G418 and are expressed with a standard deviation of at least three experiments. *P* values were calculated by Student *t*-test. Significant differences from the efficiency of GFP-Uba1 are shown by asterisks.

It was surprising that the cytoplasmic form of Uba1 may not be required for survival of tsTM3 cells at 39°C. To rescue the ts phenotype of tsTM3 cells with expression of the cytoplasmic form of Uba1, we therefore designed plasmid vectors expressing Uba1 lacking 40 amino acids residues of the N-terminus (Uba1D1-40, Fig. 4A). This particular Uba1 corresponds to Uba1B [5]. After transfection of cells with plasmid DNAs encoding GFP hybrids of the full-length or truncated form of Uba1, cells were incubated at 39°C for the appropriate number of days. Colonies on dishes were stained with methylene blue, and the mean number of colonies from triplicate dishes was calculated (Fig. 4B). Many cells formed colonies after transfection with a plasmid encoding full-length Uba1. The efficiency obtained with GFP-Uba1was two-fold higher than that obtained with Uba1-GFP, suggesting again that N-terminus fusion contributes to the effective rescue of ts defect. However, relatively few cells grew at 39°C after transfection of plasmids encoding Uba1 lacking the N-terminus hybrid (Fig. 4B). Significant differences in colony formation efficiency were found between Uba1 and Uba1D1-40 (Fig. 4C). These results suggest that the expression of Uba1B can rescue, although not effectively, the phenotype of tsTM3 cells.

### Activity of Uba1 analyzed with Fucci

**Figure 5:**
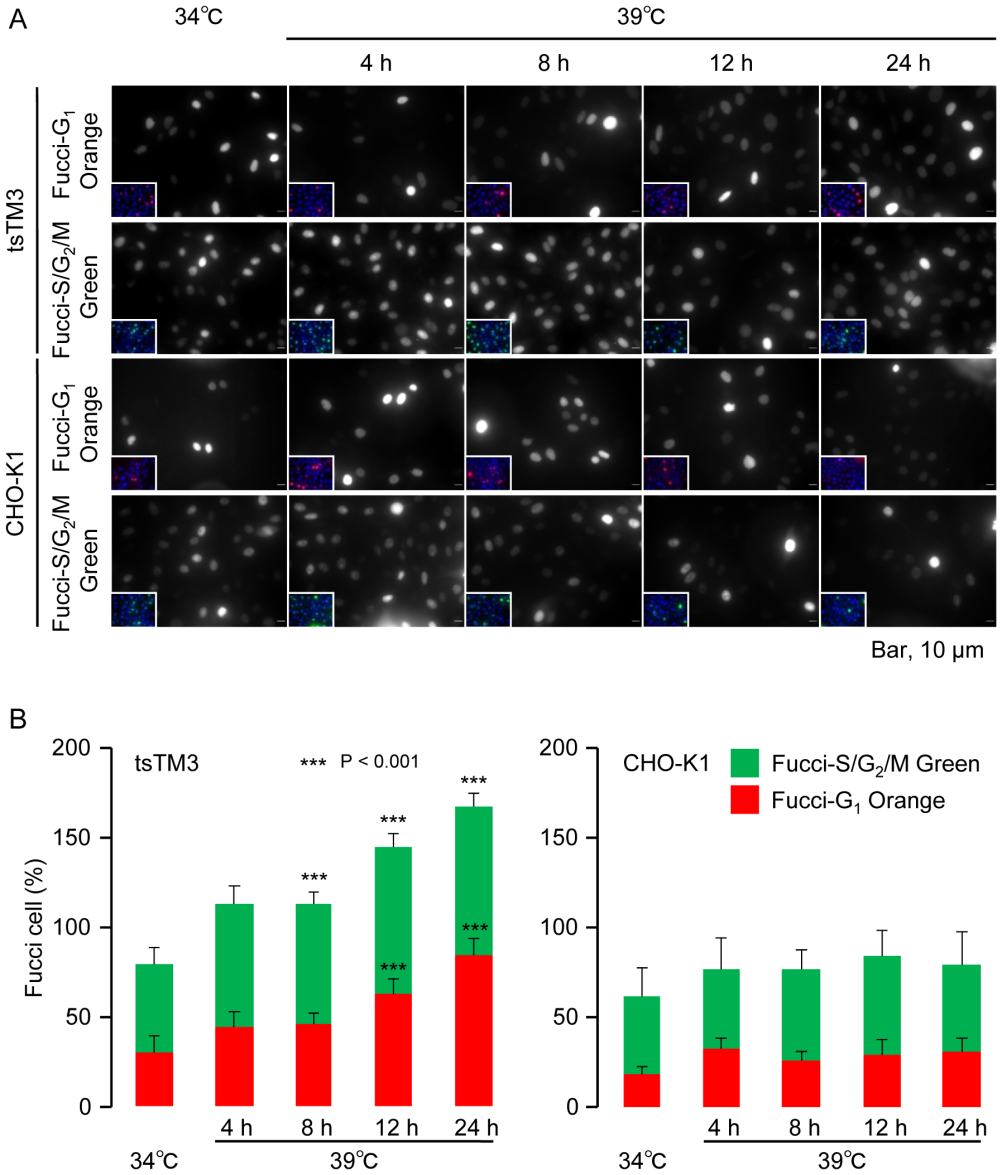
Effects of incubation at 39°C on the activity of E1 analyzed with Fucci. (A) Images of living cells expressing Fucci-G_1_ Orange or Fucci-S/G_2_/M Green in wild-type CHO-K1 or ts mutant tsTM3 cells. Stable transformants expressing Fucci were grown at 34°C or incubated at 39°C for the times shown in the figure and analyzed. Small merged images with counterstain of Hoechst 33342 are embedded. Bar, 10 µm. (B) Quantitative analyses of the numbers of cells expressing Fucci in CHO-K1 and tsTM3 cells. Cells expressing Fucci, such as those shown in panel (A), were counted and are expressed as a ratio with standard deviation of at least three experiments. *P* values were calculated by Student *t*-test. Significant differences from values at 34°C are shown by asterisks. Incubation at 39°C increased the number of tsTM3 cells expressing Fucci signals.

The results obtained from immunoblot analysis as well as the expression of Uba1 tagged with GFP showed the impaired function of Uba1 in the nucleus (Figs. 2 and 3). To analyze E1 activity in the nucleus, we investigated the changes of Fucci with live-cell imaging and Western blotting. First, we isolated cells expressing two kinds of Fucci, Fucci-G_1_ Orange and Fucci-S/G_2_/M Green, from wild-type CHO-K1 and ts mutant tsTM3 (Fig. 5A). Fucci-G_1_ Orange represents monomeric Kusabira-Orange2 (mKO2) fused to a part of human Cdt1 (mKO2-hCdt1), and Fucci-S/G_2_/M Green represents humanized monomeric Azami-Green1 (hmAG1) fused to a part of human geminin (mAG1-hGem). Only Fucci-G_1_ Orange (mKO2-hCdt1) is present in the G_1_ phase of the cell cycle, resulting in cells with red fluorescent nuclei. In the S, G_2_, and M phases, Fucci-S/G_2_/M Green (mAG1-hGem) remains as green fluorescence within the nuclei [3]. In the population of wild-type CHO-K1 cells growing at 34°C and 39°C, the proportions of cells expressing Fucci-G_1_ Orange and Fucci-S/G_2_/M Green were 18–33% and 32–55%, respectively. The proportion of CHO-K1 cells expressing Fuccis was nearly 100% and appeared to be permanent even at 39°C (Fig. 5B). A similar result was obtained from ts mutant tsTM3 cells at 34°C (Fig. 5B). However, we found significant increases of tsTM3 cells expressing each Fucci, Fucci-G_1_ Orange and Fucci-S/G_2_/M, during the incubation at 39°C (Fig. 5A). Quantitative analysis of images of living cells showed that incubation at 39°C resulted in a significant increase of tsTM3 cells expressing Fucci (Fig. 5B).

### Significant accumulation of Fucci proteins with decrease of Uba1A in tsTM3 cells at 39°C

**Figure 6:**
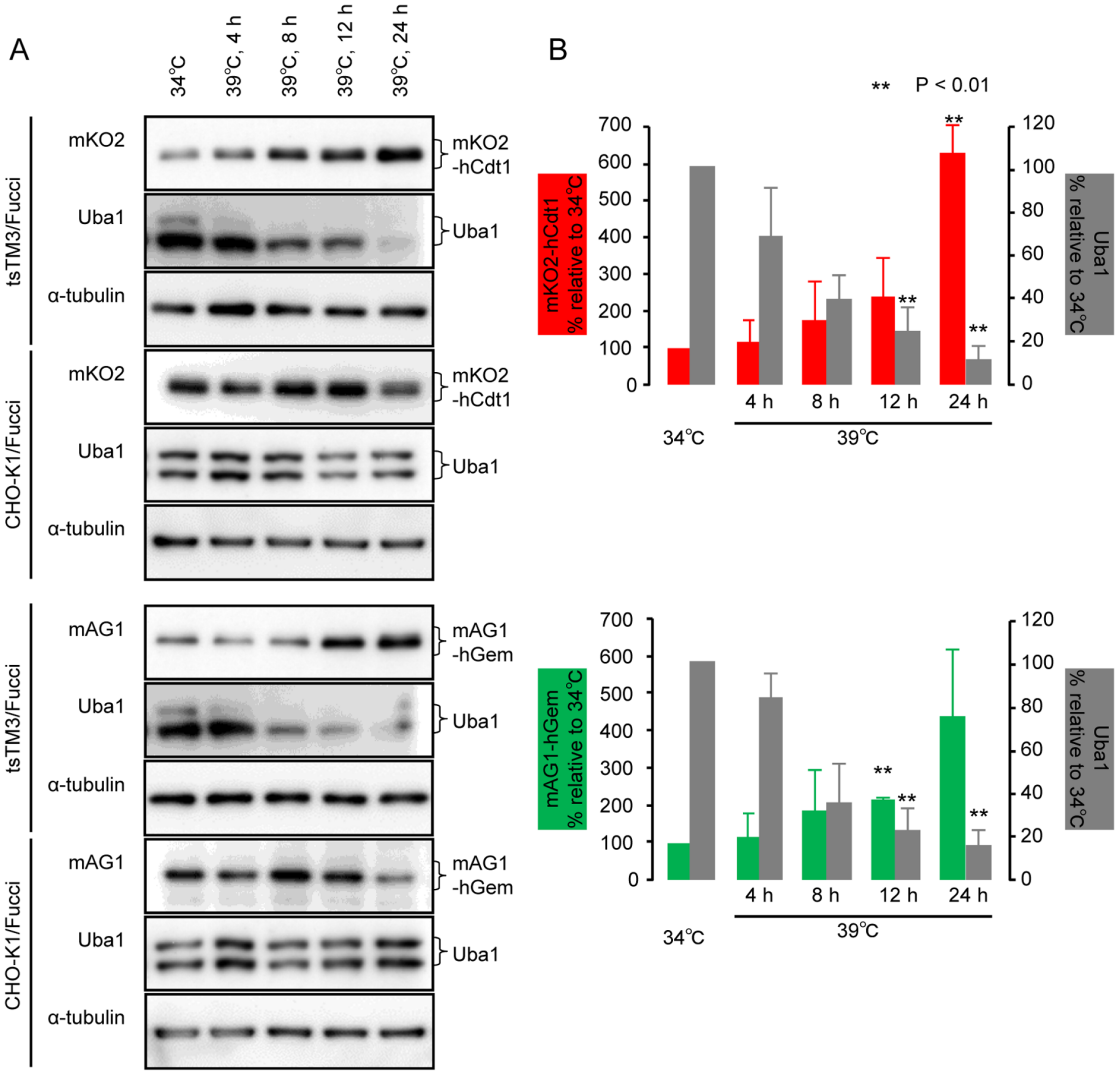
Accumulation of Fucci proteins in tsTM3 cells at 39°C. (A) Western blot analysis of Fucci in CHO-K1 or tsTM3 cells. Wild-type CHO-K1 or ts mutant tsTM3 cells expressing Fucci-G_1_ Orange or Fucci-S/G_2_/M Green were grown at 34°C or incubated at 39°C for the times shown in the figure and lysed. The proteins resolved on acrylamide gels and were detected by immunoblotting with antibodies directed against fluorescent tags (mKO2 or mAG1) and Uba1. Only relevant parts of the blot are shown. α-tubulin is included as a loading control. Temperature-dependent increases in the amount of Fucci with the reduction of Uba1 were found in tsTM3 cells. (B) Quantitative analysis of bands in blots of tsTM3 cells expressing Fucci. Band intensities of Fucci and Uba1, like those shown in panel (A), were measured and are expressed relative to that of each at 34°C or relative to the lower band at 34°C with standard deviation of at least three experiments. *P* values were calculated by Student *t*-test. Significant differences from values at 34°C are shown by asterisks. Incubation at 39°C resulted in a significant increase of Fucci (mKO2-hCdt1 or mAG1-hGem) with decrease in Uba1A in tsTM3 cells.

Next, we analyzed Fucci expressed in CHO-K1 and tsTM3 cells by Western blotting to investigate the accumulation of the Fucci proteins. Two kinds of rabbit polyclonal antibodies against each of the fluorescent proteins, mKO2 or mAG1, gave similar intensity of bands in the wild-type cells at 34°C and 39°C (Fig. 6A). This was consistent with our observation of living CHO-K1 cells expressing Fucci (Fig. 5). However, growth of the mutant tsTM3 cells at 39°C led to a significant increase in the Fucci proteins mKO2-hCdt1 or mAG1-hGem, and these increases were dependent on the length of incubation at 39°C (Fig. 6A). Uba1A had again decreased gradually and significantly in mutant cells at 39°C (Fig. 6A), and again, this was consistent with the result shown in Figure 2. The 8-hour incubation of tsTM3 cells at 39°C resulted in an approximately two-fold increase in Fucci proteins, and the quantity of Uba1 decreased by about one half (Fig. 6B). After 24 hours of incubation at 39°C, quantities of mKO2-hCdt1 and mAG1-hGem in tsTM3 cells had increased by approximately 6.2- and 4.4-fold, respectively, compared with those at 34°C (Fig. 6B), and little Uba1A was detected (Fig. 6A, B). The significant increase in Fucci proteins appears to be inversely proportional to the decrease in Uba1 protein, especially that of the large form. These results suggest that loss of function of Uba1 in the nucleus may lead to failure of the Ub system followed by accumulation of proteins, which should be degraded properly by the Ub-proteasome system in the usual manner.

### Significant accumulation of endogenous geminin in tsTM3 cells at 39°C

**Figure 7:**
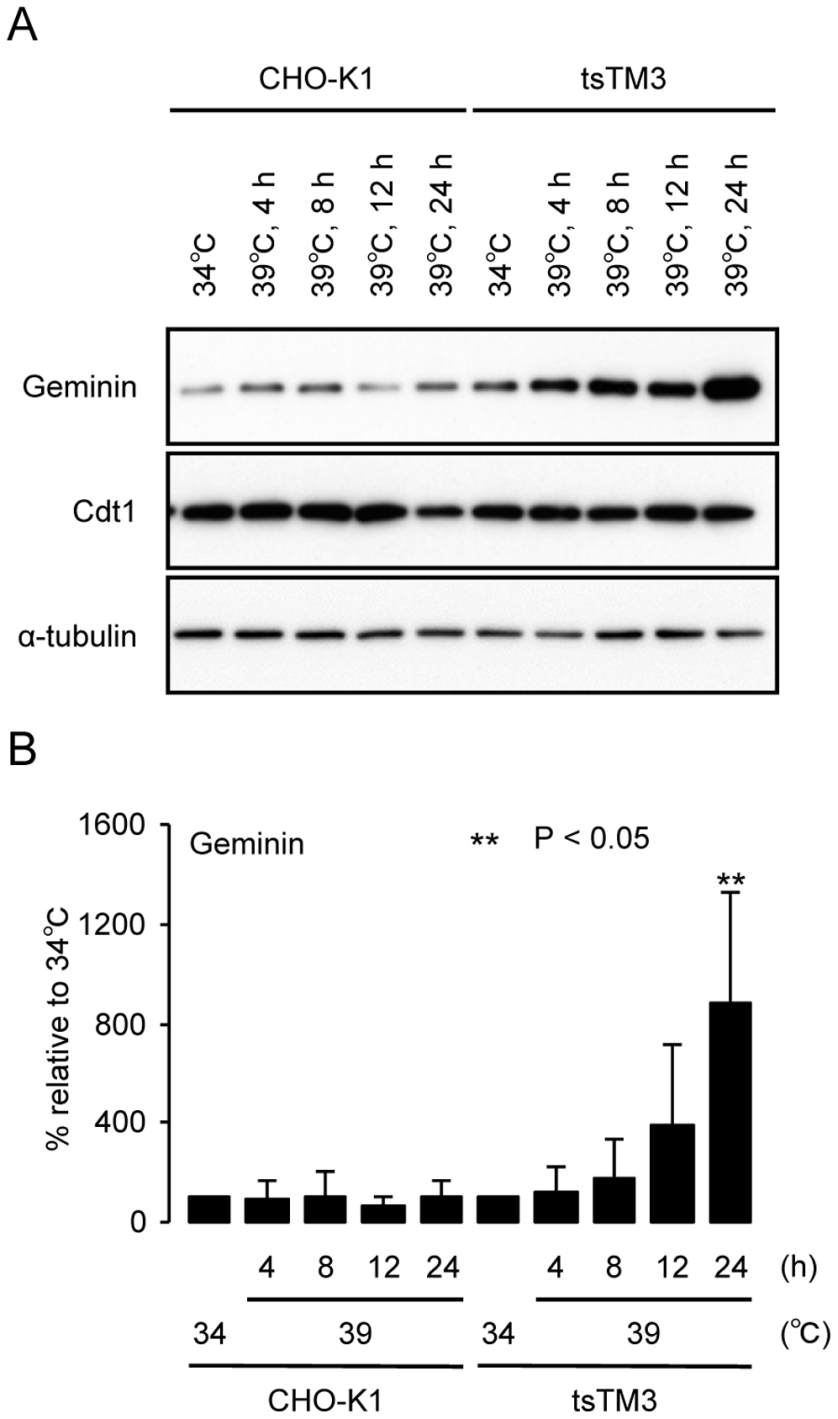
Effect of ts defect of Uba1 on endogenous proteins. (A) Western blot analysis of endogenous geminin and Cdt1. Wild-type CHO-K1 or ts mutant tsTM3 cells were grown at 34°C or incubated at 39°C for the times shown in the figure and lysed. The proteins resolved on acrylamide gels and were detected by immunoblotting with antibodies directed against geminin and Cdt1. Only relevant parts of the blot are shown. α-tubulin is included as a loading control. Temperature-dependent increase in geminin, but not in Cdt1, was found in tsTM3 cells. (B) Quantitative analyses of the amounts of geminin in CHO-K1 and tsTM3 cells. Band intensities, such as those shown in panel (A), were measured and are expressed relative to that of each at 34°C with standard deviation of at least three experiments. *P* values were calculated by Student *t*-test. Significant differences from values at 34°C are shown by asterisks. Incubation at 39°C resulted in a significant accumulation in geminin in tsTM3 cells.

We then analyzed endogenous geminin and Cdt1 proteins in tsTM3 cells by Western blotting because both Fucci proteins mKO2-hCdt1 and mAG1-hGem are derived from Cdt1 and geminin, respectively, which are known to be crucial regulators of DNA replication. Antibodies against geminin and Cdt1 gave similar intensity of bands in the wild-type cells at 34°C and 39°C (Fig. 7A). However, growing the ts mutant tsTM3 cells at 39°C led to a significant increase in the geminin, and these increases were dependent on the length of incubation at 39°C (Fig. 7A). Interestingly, little accumulation of Cdt1 was observed in the tsTM3 cells. The 12-hour incubation of tsTM3 cells at 39°C resulted in an approximately four-fold increase in geminin (Fig. 7B). After 24 hours of incubation at 39°C, the quantity of geminin in tsTM3 cells had increased by approximately 8.8-fold compared with that at 34°C (Fig. 7B).

**Figure 8:**
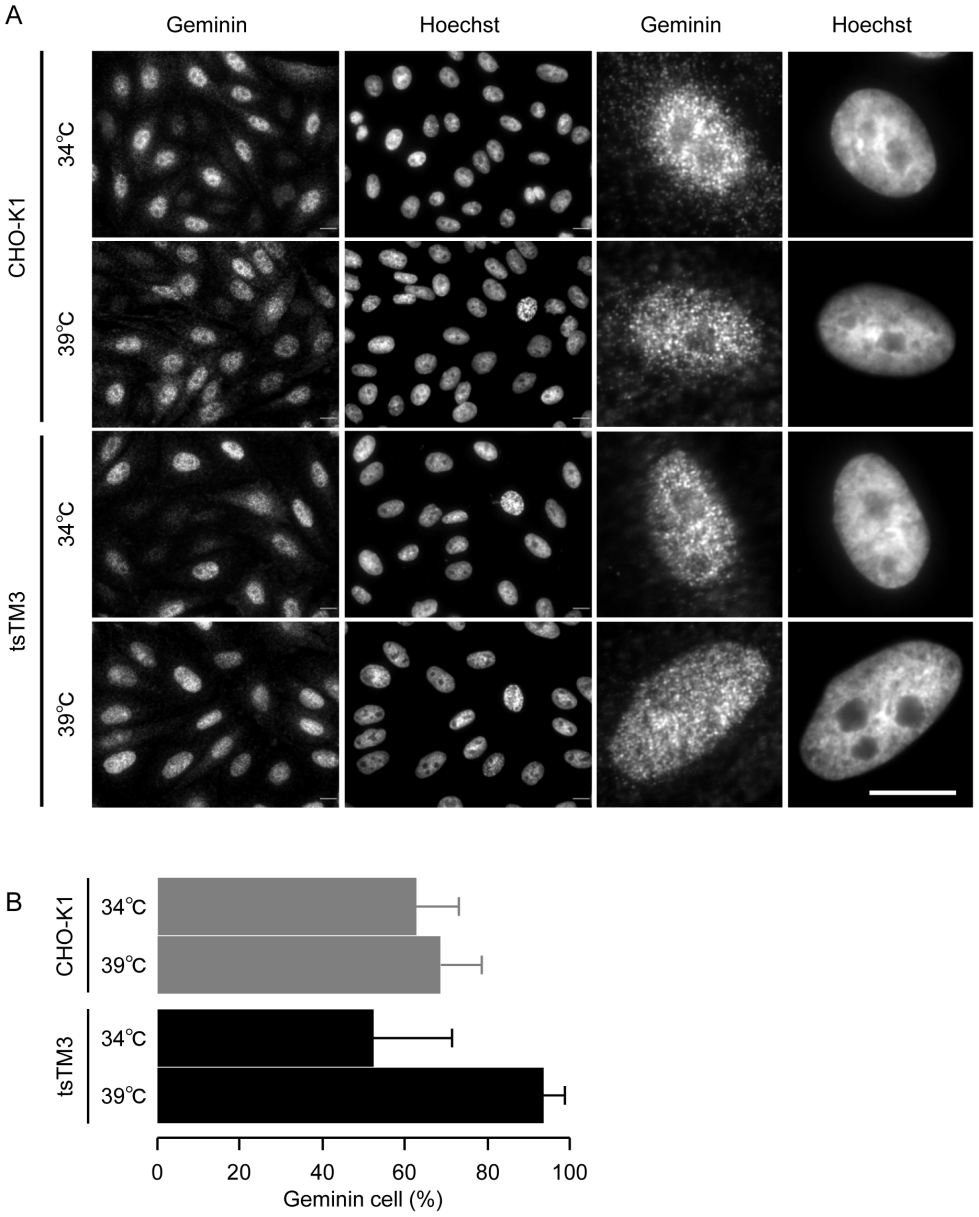
Incubation at 39°C led to an increase in endogenous geminin-positive tsTM3 cells. (A) Wild-type CHO-K1 or ts mutant tsTM3 cells were grown at 34°C or incubated at 39°C for 12 hours and fixed. Next, endogenous geminin was indirectly immunolabeled with Alexa 594, and cells were counterstained with Hoechst 33342. Scale bar, 10 µm. Higher magnification views are shown to the right of the low-power images. Incubation at 39°C led to an increase in geminin-positive tsTM3 cells. Localization of geminin to the nucleolus was found in tsTM3 cells at 39°C. (B) Quantitative analyses of the numbers of cells expressing geminin in CHO-K1 and tsTM3 cells. Cells expressing geminin, like those shown in panel (A), were counted and are expressed as a ratio with standard deviation of at least three experiments. Incubation at 39°C increased the number of tsTM3 cells expressing geminin, although differences from values at 34°C were not significant.

We next investigated the effect of the ts defect of Uba1 on the distribution of geminin in tsTM3 cells by indirect immunolabeling. A rabbit polyclonal antibody against geminin gave similar patterns in the wild-type cells at 34°C and 39°C and in the mutant cells at 34°C, yielding many small bright foci in discrete nuclear sites (Fig. 8A). This was consistent with our observation of cells expressing mAG1-hGem (Fig. 5A). In the population of wild-type CHO-K1 cells growing at 34°C and 39°C, the proportions of cells expressing geminin were 62.6% and 68.6%, respectively (Fig. 8B). A similar result was obtained from ts mutant tsTM3 cells at 34°C (Fig. 8B), but we found an increase of tsTM3 cells expressing geminin during the incubation at 39°C (Fig. 8A). Expression of geminin appeared to be present in almost all of the mutant cells at 39°C, although quantitative analysis showed no statistically significant difference with that at 34°C (Fig. 8B). These results from Western blotting and indirect immunolabeling suggest that loss of function of Uba1 results in an accumulation of endogenous geminin in tsTM3 cells during incubation at 39°C.

### Cdt1 remained in active replication sites in tsTM3 cells incubated at 39°C

**Figure 9:**
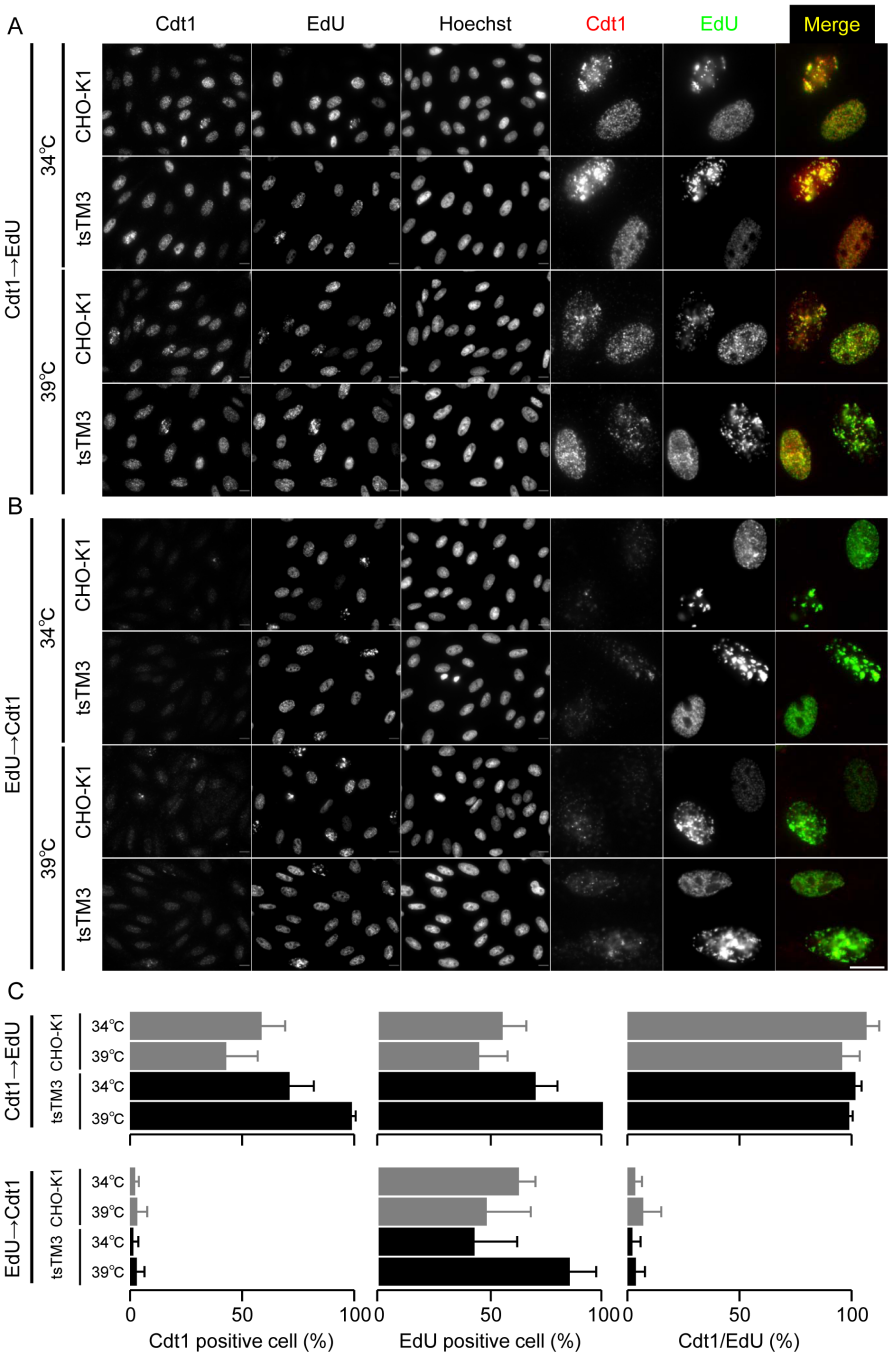
Cdt1 remained in active replication sites in tsTM3 cells incubated at 39°C. Cells were grown on glass coverslips at 34°C or 39°C for 12 hours, incubated with 10 µM EdU for 20 minutes, washed, and fixed with 4% PFA. (A) Cdt1 was indirectly immunolabeled with Alexa 594. Next, nascent DNA labeled with EdU was detected with Alexa 488 by Click-iT reaction, and cells were also counterstained with Hoechst 33342. The merged views (right) are composed of Cdt1 (red channel) and EdU (green channel). Cdt1 was found in many discrete nuclear sites, and most of these also contained EdU, which results in the yellow color in the merged images, indicating co-localization between Cdt1 and nascent DNA. (B) Nascent DNA labeled with EdU was detected with Alexa 488 by Click-iT reaction. Next, Cdt1 was indirectly immunolabeled with Alexa 594. Labeling of Cdt1 was significantly decreased, and little yellow color was found in the merged images. Bar, 10 µm. (C) Quantitative analyses of the numbers of cells expressing Cdt1 and EdU incorporated cells. Cells expressing Cdt1 and cells labelled with EdU, such as those shown in panels (A) and (B), were counted and are expressed as a ratio with standard deviation in the left and middle graphs, respectively. The proportion of cells expressing Cdt1 with cells labelled with EdU is shown in the right graph.

Western blot analysis showed the retention of Cdt1 in the tsTM3 cells during the incubation at 39°C, although little accumulation of Cdt1 was observed (Fig. 7A). We therefore investigated the effect of the retention of Cdt1, especially with regard to the link to DNA synthesis, and performed sequential double staining for Cdt1 and nascent DNA. Nascent DNA was labeled by a thymidine analogue, 5-ethynyl-2′-deoxyuridine (EdU), and detected by Click-iT reaction with fluorescent dye. First, after indirect immunolabeling of Cdt1, detection of EdU was carried out sequentially. A rabbit polyclonal antibody against Cdt1 yielded many small and some large bright foci in discrete nuclear sites and gave similar patterns in the wild-type cells at 34°C and 39°C and in the mutant cells at 34°C (Fig. 9A). We found Cdt1 in the nucleus of tsTM3 cells after 12 hours of incubation at 39°C, suggesting the retention of Cdt1. Most of these cells contained nascent DNA labeled by EdU, which results in the yellow color in the merged images, indicating co-localization between Cdt1 and nascent DNA (Fig. 9A and C). These results are consistent with a previous finding that DNA synthesis in tsTM3 cells is still active after 10 hours of incubation at 39°C [8]. Next, after fluorescent labeling of nascent DNA by Click-iT reaction, endogenous Cdt1 was indirectly immunolabeled. Nascent DNA was found again in many small and some large bright foci in discrete nuclear sites (Fig. 8B). However, labeling of Cdt1 was significantly decreased, and little yellow color was found in the merged images (Fig. 9B and C). Labelling of nascent DNA appears to inhibit indirect immunolabeling for Cdt1, raising the possibility that the physical distance between Cdt1 and nascent DNA may be very close. These results indicate that Cdt1 remained in active replication sites in tsTM3 cells incubated at 39°C.

## Discussion

### Temperature-sensitive mutants for Uba1

In this study, we identified a point mutation in *Uba1* isolated from the ts mutant tsTM3 cells, which led to a Met-to-Ile substitution at amino acid 256 in deduced Uba1 protein. Characterization of this mutation revealed that a Met-to-Ile substitution at amino acid 256 is the major cause of Uba1 instability, especially in the nucleus. Many Uba1 mutants have been isolated from several cell lines, and some earlier studies showed a reduction of Uba1 at the restricted temperature, although these studies did not distinguish the nuclear form from total Uba1 [22]–[24]. We found that incubation of tsTM3 cells at 39°C resulted in a deficiency of nuclear Uba1 and in impaired ubiquitination in the nucleus.

### Mutations found in *Uba1*

It may be possible for the amino acid substitution of Uba1 found in tsTM3 cells to affect substrate recognition and enzyme activity because the mutated methionine residue was also localized in the catalytic domain. Analysis with the mouse ts mutant cell line ts20 characterized two mutations that impair the stability and function of Uba1 [21]. These mutations led to two amino acid substitutions, A189T and W719C (Fig. 1B), resulting in instability of the Uba1 protein. A ts mutation W719C in ts20 cells was located in the SCCH. The instability of Uba1 is consistent with our results from immunoblot analysis of tsTM3 incubated at the non-permissive temperature. For the important amino acid residue in the FCCH characterized at present, cystain-278 in FCCH is sensitive to oxidation and can affect Ub charging through a change in its proximity to Ub [25], although the mutation found in tsTM3 cells is not close to the mutations listed above.

### Nuclear and cytoplasmic subpopulations of Uba1

The rescue efficiency of tsTM3 cells at 39°C by the expression of GFP-Uba1 was higher than that by the expression of Uba1-GFP. Fluorescent microscopy and Western blot analyses showed that most of GFP-Uba1 localized in the nucleus, although we cannot exclude the possibility of the cytoplasmic localization of Uba1A. We think that GFP fusion to the N-terminus of Uba1 may function to prevent the sequence of nuclear localization from undergoing alternative translation or alternative splicing because the first 11 amino acids are essential for exclusive nuclear localization of Uba1 and the second methionine residue located downstream of this nuclear localization sequence at amino acid position 41 [26]. It should be noted that although few in number, obvious colonies of tsTM3 cells at 39°C were observed after transfection of plasmids encoding Uba1 lacking 40 amino acids at the N-terminus. The precise mechanism of this rescue remains unclear, but it is possible that retention of the cytoplasmic form of Uba1 in the nucleus is caused by mitosis or by shuttling.

### Restricted role of Uba1 in the nucleus

Incubation at the non-permissive temperature of derivatives of tsTM3 cells expressing Fuccis resulted in an accumulation of short-lived proteins through the cell cycle, suggesting impaired ubiquitination in the nucleus by the ts defect of Uba1. A potential role for Uba1 in turnover of nuclear protein was predicted from the fact of its nuclear localization [27], [28]. Accumulation of endogenous p53 protein was found in another Uba1 ts mutant mouse cell line, ts20 [29]. We also showed an accumulation of endogenous geminin in tsTM3 cells. Further, an earlier study showed substrate-specific ubiquitination and degradation with requirements of a different amount of Uba1 in these mouse ts20 cells [24]. Our immunoblot analyses showed that a significant decrease of Uba1 in the nucleus may lead to the ts defects seen in tsTM3 cells incubated at 39°C. Judging from the analyses of derivatives of GFP-Uba1, which presented with a single large form of GFP-tagged Uba1, we think that the expression of Uba1A is adequate to rescue tsTM3 cells at 39°C. These results also suggest that ubiquitination levels in the nucleus are strongly related to Uba1 activity and that Uba1 Met-256 appears to affect the role of Uba1 in the nucleus but not in the cytoplasm. A recent study identified Uba1, but not Uba6, as the critical enzyme essential for the formation of radiation-induced foci, timely DNA repair, and for response to replication stress [30]. This is consistent with our results.

### Uba1 and its relation to the maintenance of genome integrity

We found that incubation at 39°C resulted in an accumulation of Fuccis in mutant cells, suggesting that degradation of geminin and Cdt1 by the Ub-proteasome system was impaired. Western blot analysis also revealed that incubation at 39°C caused an accumulation of endogenous geminin in mutant cells. The quantity of endogenous Cdt1 in tsTM3 cells appeared to be permanent even after incubation at 39°C, although we found an accumulation of Fucci mKO2-hCdt1, which is a Cdt1 derivative. The discrepancy in the results between Fucci and endogenous Cdt1 protein appears to be explained by the differences in their expression in tsTM3 cells arrested in the S to G_2_ phases of the cell cycle. Retention of Cdt1 appears to contribute to the retention of activity of DNA synthesis at 39°C. However, the dominant quantity of geminin to that of Cdt1 might contribute to the inhibition of DNA replication. Judging from the results in this study, inhibition of DNA replication may require an incubation at 39°C of longer than 12 hours. DNA replication is highly regulated by a number of licensing and replication factors during the cell cycle. Loss of proper control leads to deregulated DNA replication, which causes genome instability. In mammalian cells, both Cdt1 and its inhibitor geminin are crucial regulators of this orchestrated licensing of DNA replication [31]. In tsTM3 cells, it is possible that a mutation of *Uba1* causes the defect in licensing of DNA replication, which in turn leads to chromosome instability and cell-cycle arrest. Recent studies have shown that the balance of Cdt1:geminin appears to be crucial for regulation of proper replication in somatic cells and *Xenopus* embryos, especially by relying on changing interactions between Cdt1 and geminin during the cell cycle but not their degradation [32], [33]. It appears to be difficult to explain the complicated phenotypes found in tsTM3 cells solely by the accumulation of geminin and the retention of Cdt1. However, phenotypes in DNA replication may be caused by an inappropriate balance between Cdt1 and geminin.

We conclude that the ts phenotype of tsTM3 cells is determined by a G-to-A transition at nucleotide 768 of *Uba1* that results in a Met-to-Ile substitution at amino acid 256 in the catalytic domain of Uba1. At the nonpermissive temperature, mutant cells showed significant decrease of Uba1, especially in the nucleus, and ubiquitination activity in the nucleus decreased significantly. Complementation test with Uba1 tagged with GFP revealed that nuclear localization of Uba1 appears to be adequate to rescue tsTM3 cells at the nonpermissive temperature. Taken together with previous observations, these results suggest that a Met-to-Ile substitution at amino acid 256 in Uba1 affects ubiquitination in the nucleus, resulting in an inappropriate balance between Cdt1 and geminin followed by defects in DNA synthesis. The impaired modulation of DNA replication may give rise to cell-cycle arrest in the S to G_2_ phases and to chromosome instability.

## Materials and Methods

### Cells and microscopy

Cells were grown in Ham's F-12 medium (Sigma-Aldrich, St. Louis, MO) containing 10% fetal calf serum, 2 mM L-glutamine, and antibiotics (Gibco/Invitrogen, Carlsbad, CA) at 34°C. For the analysis of ts phenotype, cells were shifted up to the nonpermissive temperature (39°C). Images of cells growing in glass-bottom culture dishes were collected with an Olympus DP30BW digital charge-coupled device camera fitted on an Olympus IX71 microscope (Olympus, Tokyo, Japan) and “contrast-stretched” with Adobe Photoshop CS (Adobe Systems, San Jose, CA).

### Cloning and sequencing

The sequences of hamster *Uba1* from wild-type and mutant cells were determined by using a strategy described previously [9]. In brief, mRNAs were prepared from the two cell lines and reverse-transcribed to cDNAs. The resulting cDNAs were amplified with the primer pair UBA15b, 5′-GAAGATCTATGTCCAGCTCGCCGCTGTC-3′ and UBA13s, 5′-GCCGACGTCGACGCGAATGGTATATCGGACAT-3′. PCR products were then analyzed by direct sequencing with the above primer pair and with six additional primers: UBA152, 5′-AAAGTGCTGGGTCCTTACAC-3′; UBA152C, 5′-GTGTAAGGACCCAGCACTTT-3′; UBA132, 5′-GAACTTGTACGGACTGCAGA-3′ and UBA132C, 5′-TCTGCAGTCCGTACAAGTTC-3′; UBA153, 5′-TAAAGTCTGACACAGCCGCT-3′; and UBA133, 5′-TCATCTGTCGAACAGCTGCA-3′. At least three independent mRNA samples were prepared from both wild-type and mutant cells. Genomic DNA from the two cell lines was also amplified with the primer pair UBA154, 5′-GACAATCCCGGTGTGGTTAC-3′ and UBA134, 5′-GTTGGAGGTGTCACAGATAC-3′ and sequenced directly by the same approach.

### Plasmids and transfection

The *Bgl*II-*Sal*I restriction fragment containing the coding sequence of hamster *Uba1* cDNA was cloned into the vectors pEGFP-C1 and pEGFP-N3 (Clontech Laboratories, Palo Alto, CA) to yield the plasmids GFP-Uba1 and Uba1-GFP, respectively. The truncated form of *Uba1* cDNA was amplified with the primer pair UBA1B5b, 5′-GAAGATCTATGGCGAAGAACGGCAGTGA-3′ and UBA13s and was cloned into the vectors pEGFP-C1 and pEGFP-N3 (Clontech Laboratories) to yield the plasmids GFP-Uba1D1-40 and Uba1D1-40-GFP, respectively. Plasmids (2 µg) encoding GFP hybrids were introduced into tsTM3 cells with the FuGENE6 transfection reagent (Roche Diagnostics, Mannheim, Germany), and clones able to grow at 39°C were selected. Colonies in dishes were stained with methylene blue and counted after selection at 39°C or 34°C with 400 µg/ml G418 (Roche Diagnostics), and the average colony number for triplicate dishes was calculated.

Two expression vectors of the Fucci, pFucci-G_1_ orange and pFucci-S/G_2_/M green (Medical and Biological Laboratories [MBL], Nagoya, Japan), were introduced into CHO-K1 or tsTM3 cells with the FuGENE6 transfection reagent and selected with 400 µg/ml G418 (Roche Diagnostics).

### Antibodies and immunoblotting

We measured the quantity of Uba1 in tsTM3 cells by immunoblotting because we previously have characterized a significant decrease in causative protein Smu1 in another ts-mutant, tsTM18 cells [34]. Approximately 10^6^ cells in a culture dish were lysed by adding sample buffer containing sodium dodecylsulfate (SDS) and heating at 95°C for 10 minutes. Proteins from 10^3^ cells were resolved on 6% or 12% SDS-polyacrylamide gels, and target proteins were detected by immunoblotting [35]. Primary antibodies used in this study were two kinds of rabbit anti-Ub activating enzyme E1 (1:1000; Rockland, Gilbertsville, PA, and 1:1000; Calbiochem, La Jolla, CA), rabbit anti-monomeric Kusabira-Orange 2 (mKO2, 1:1000; MBL), rabbit anti-monomeric Azami-Green 1 (mAG1, 1:1000; MBL), rabbit anti-GFP (1:500; MBL), mouse anti-α-tubulin (clone DM 1A, 1:500; Sigma-Aldrich), rabbit anti-SMC3 (1:250; Abcam, Cambridge, UK), rabbit anti-geminin (1:1000; Sigma-Aldrich), and rabbit anti-Cdt1 (clone EPR4729, 1:1000; Abcam). Digital images were collected by scanning the film, and band intensities were measured with ImageJ 1.34S (http://rsb.info.nih.gov/ij/).

### EdU labelling and indirect immunolabeling

Cells were grown on glass coverslips at 34°C or 39°C for 12 hours, incubated with 10 μM 5-ethynyl-2′-deoxyuridine (EdU) for 20 minutes, and washed and fixed for 20 minutes at 4°C with 4% paraformaldehyde (PFA) in 250 mM HEPES. Labelled DNA was detected with Alexa Fluor 488 using “Click-iT” detection reaction according to the manufacturer's instructions (Invitrogen/Molecular Probes, Eugene, OR).

Procedures for the indirect immunolabeling and microscopy of immunolabeled cells have been described previously [36]. Primary antibodies used in this study were rabbit anti-geminin (1:800; Sigma-Aldrich) and rabbit anti- Cdt1 (1:800; Sigma-Aldrich).
